# Impacts of Organic and Conventional Crop Management on Diversity and Activity of Free-Living Nitrogen Fixing Bacteria and Total Bacteria Are Subsidiary to Temporal Effects

**DOI:** 10.1371/journal.pone.0052891

**Published:** 2012-12-28

**Authors:** Caroline H. Orr, Carlo Leifert, Stephen P. Cummings, Julia M. Cooper

**Affiliations:** 1 Faculty of Health and Life Sciences, Northumbria University, Newcastle-Upon-Tyne, United Kingdom; 2 Nafferton Ecological Farming Group, Newcastle University, Nafferton Farm, Stocksfield, Northumberland, United Kingdom; U. S. Salinity Lab, United States of America

## Abstract

A three year field study (2007–2009) of the diversity and numbers of the total and metabolically active free-living diazotophic bacteria and total bacterial communities in organic and conventionally managed agricultural soil was conducted using the Nafferton Factorial Systems Comparison (NFSC) study, in northeast England. Fertility management appeared to have little impact on both diazotrophic and total bacterial communities. However, copy numbers of the *nifH* gene did appear to be negatively impacted by conventional crop protection measures across all years suggesting diazotrophs may be particularly sensitive to pesticides. Impacts of crop management were greatly overshadowed by the influence of temporal effects with diazotrophic communities changing on a year by year basis and from season to season. Quantitative analyses using qPCR of each community indicated that metabolically active diazotrophs were highest in year 1 but the population significantly declined in year 2 before recovering somewhat in the final year. The total bacterial population in contrast increased significantly each year. It appeared that the dominant drivers of qualitative and quantitative changes in both communities were annual and seasonal effects. Moreover, regression analyses showed activity of both communities was significantly affected by soil temperature and climatic conditions.

## Introduction

Yields of arable crops depend on sufficient reservoirs of plant available nitrogen in agricultural soils. However, as conventional fertility management using inorganic nitrogen fertiliser is becoming increasingly expensive and is recognised as having significant detrimental effects on the environment [1], there is growing interest in more sustainable systems that can exploit biologically fixed nitrogen or use inorganic nitrogen as efficiently as possible.

One microbiological approach to improve sustainability is to enhance the activity of the nitrogen fixing bacteria in soil [2] and to optimise the effects of land use [3], crop management [4], [5], N management [4], and seasonal variations [6] on N fixation processes, especially by free-living diazotrophs.

Fertility management, crop protection and crop rotation have all been shown to exert significant effects on the soil microbial communities present in organically and conventionally managed agricultural soils. Previous studies that looked at the impact of farm management on the function and diversity of these communities report the most significant factor affecting soil microbial communities is the fertility management regime [7], [8]. However, results are equivocal and studies have mostly focussed on comparing farming systems over a single season. Here we extend these studies by exploring the effects of different organic and conventional farm management practices on the total bacterial and free-living N fixing community using a factorial design that allows us to investigate the individual effects of crop protection practices and fertility management over several seasons. In general, organic fertility management systems, that include the application of farmyard manure, and the use of diverse crop rotations have been shown to have a positive effect on microbial biomass, diversity and activity [9], [10], [11], [12], when compared with conventional systems. These differences are mainly attributed to; the increased organic C added as manure; lower background levels of readily-available nitrogen, and pH values that are, on average, closer to neutral in organically managed soils [13], [14]. As nitrogen fixation is energy-expensive, it is reliant on carbon sources that are more abundant and are retained longer in organically managed compared to conventional soils [15]. Therefore, organic soils are more likely to offer optimal conditions for nitrogen fixation and it is perhaps unsurprising that increases in soil organic carbon have been shown to stimulate nitrogen fixation, although results have been inconsistent [16], [17], [18], [2]. Additional drivers of the activity of the diazotrophic community have been identified as the soil microbial biomass and total nitrogen [3] both of which are typically higher in organic systems.

Other secondary effects of fertility management could also be significant, in particular, changes in soil pH, which is considered a predictor of soil microbial community composition [19]. Hallin et al, [20] found that pH affected total bacterial community composition among soils treated with different fertilizers. Phosphorus can also stimulate nitrogen fixation as it is required for microbial energy production. Reed et al, [21] observed doubling of nitrogen fixation in response to the addition of phosphorus. Most studies discussing free-living N fixing bacteria have described results from a single season (e.g. [22], [23]. Since free-living N fixers are known to be very sensitive to environmental conditions, it is important to establish whether crop management effects are consistent across dates within a given year, and over several growing seasons.

Crop protection measures could also potentially affect the soil microbial community. Conventional farmers can use a complex mixture of pesticides to protect their crops [24]; whereas, organic farmers rely on the diversification of crops in the field (intercropping) and over time (crop rotation), use of resistant varieties, optimal timing of tillage for weed control, and use of a limited range of organically approved pesticides [25]. While conventional crop protection measures include the use of chemicals that are toxic to specific organisms, the majority of studies into the effects of chemical pesticides on the soil microbial community have found that they do not significantly affect microbial populations when used at the correct dose [26], [27], [28]. Nitrogen fixing bacteria are thought to be especially sensitive to pesticides [29]. However, most of the work looking at the effects of pesticides has been carried out on symbiotic diazotrophs. For example, it was shown both *in vitro* and *in vivo* that around 30 different pesticides have a negative effect on the relationship between *S. meliloti* and alfalfa probably due to a disruption in the chemical signalling between the bacteria and its host [30], [31].

In this study we utilise an existing factorial field trial that enables comparisons between elements of organic and conventional systems, including fertility management, crop protection and crop rotation, to be analysed over several years. Previously, we have used this trial to demonstrate how organic and conventional crop rotations affect the bulk soil microbial community with emphasis on free-living diazotrophs [23] within a single growing season. In this paper the effects of fertility management and crop protection as well as sample date and sample year, on the general total bacterial and diazotrophic communities over three years are reported.

## Materials and Methods

### Soil Sampling

The soil (sandy loam; Stagnogley) used in this study was taken from the Nafferton Factorial Systems Comparison (NFSC) study, a field trial based at Nafferton Farm in the Tyne Valley, northeast England. The NFSC was established in 2001 and consists of a series of four field experiments established within four replicate blocks. Each experiment is a split split-plot design with three factors. The main factor is crop rotation: an eight year, conventional cereal intensive rotation is compared to an eight year, diverse legume intensive organic crop rotation. Each crop rotation main plot is split to compare two levels of crop protection: organic (ORG CP; according to Soil Association organic farming standards [32]) and conventional (CON CP; following British Farm Assured practice). Each crop protection subplot is further split into two fertility management sub-subplots: organic (ORG FM) and conventional (CON FM). Each of the four field experiments has the same design, but was begun in a different year to allow a diversity of crops to be grown in the trial in any given year. In this study soils were taken from potato plots (6 x 24 m in size) grown in 2007, 2008 and 2009 on three dates in each year (March, July, September). Soil samples in March were taken from bare soil which had been mouldboard ploughed the previous autumn, prior to the application of any fertility or crop protection treatments. Before samples were taken in June, the soil had undergone secondary tillage and potato planting, as well as frequent ridging for weed control. Pesticides had been applied to CON CP treatments and mineral fertilisers to CON FM treatments. Compost was applied to ORG FM treatments in April prior to potato planting. Final samples were taken post harvest. Prior to potato harvest CON CP treatments were treated with a chemical defoliant, while ORG CP plots were mechanically defoliated. All potato crops followed a winter cereal the previous year; however, in 2007, the potatoes were in a conventional crop rotation following a crop of winter barley that followed two previous years of winter wheat following a grass/clover ley. In contrast, both the 2008 and 2009 potato crops were grown in an organic crop rotation following a preceding crop of winter wheat that followed a grass/clover ley.

Full details of the organic and conventional fertility management and crop protection practices used in the potato crop and for the preceding year are shown in Table 1.

**Table 1:** Crop protection protocols and fertility management used in the NFSC experiments for 2006, 2007, 2008 and 2009 under organic crop protection (ORG CP) or conventional crop protection (CON CP) and organic fertility management (ORG FM) or conventional fertility management (CON FM).

Current crop	
Potatoes (2007–9)	
ORG CP	mechanical weeding (ridging); copper-oxychloride^b^ (23 kg/ha)
CON CP	aldicarb^d^ (33.5 kg/ha); linuron^a^ (3.5 L/ha); fluazinam^c^ (1.5 L/ha); mancozeb and metalaxyl-M^c^ (4.7 kg/ha); oiquat^e^ (2 L/ha)
ORG FM	composted cattle manure (equivalent to 180 kg total N/ha with 2–9% of total N in plant available forms; 2–17 kg total P_2_O_5_/ha; 5–149 kg total K_2_O/ha)
CON FM	0∶20:30 (134 kg P_2_O_5_/ha; 200 kg K_2_O/ha); Nitram (180 kg N/ha)
**Previous crop**	
Winter barley (2006)	
ORG CP	mechanical weeding (finger weeder)
CON CP	Pendimethalin^a^ (2.5 L/ha); isoproturon^a^ (1.5 L/ha); Duplosan^a^ (1 L/ha); Acanto^b^ (0.4 L/ha); Proline^b^ (0.4 L/ha); Corbel^b^ (0.5 L/ha); Fluroxypyr^b^ (0.75L/ha); Amistarb (0.25 L/ha); Bravo 500^b^ (0.5 L/ha); Cleancrop EPX^b^ (0.4 L/ha)
ORG FM	no amendment
CON FM	0∶20:30 (64 kg P2O5/ha; 96 kg K2O/ha); Nitram (170 kg N/ha)
Winter Wheat (2007–2008)	
ORG CP	mechanical weeding (finger weeder)
CON CP	isoproturon^a^ (6 L/ha); Optica^a^ (1 L/ha); Pendimethalin^a^ (1.5 L/ha); Corbel^b^ (0.2 L/ha); Cleancrop EPX^b^ (1.25 L/ha); Bravo 500^b^ (1.75 L/ha); chlormequat^c^ (2.3 L/ha); Tern^b^ (0.15 L/ha); Twist^b^ (0.25 L/ha)
ORG FM	no amendment
CON FM	0∶20:30 (64 kg P2O5/ha; 96 kg K2O/ha); Nitram (210 kg N/ha)

a herbicide;

b fungicide;

c growth regulator;

d nematicide;

e desiccant.

Soils were sampled and a standard set of soil properties (pH, soil organic C, soil total N, NO_3_-N, NH_4_-N, soil basal respiration (SBR) and Mehlich 3-extractable P, K, and Fe) were analysed as described in Orr *et al,* [23]. Weather conditions at the experimental site were monitored on an hourly basis using a Delta-T GP1 datalogger with sensors. Mean results for soil temperature and rainfall in the 14 days prior to each soil sampling occasion are shown in Table S1.

### Nucleic Acid Extraction and PCR

RNA was extracted from 0.25 g of soil with the UltraClean microbial RNA isolation kit (MoBio) and reverse transcribed with the Superscript II reverse transcriptase kit (Invitrogen). DNA was extracted from 0.25 g of soil using the UltraClean Soil DNA extraction kit (MoBio). The *nifH* gene was amplified using an adapted method first described by Wartiainen et al [33]. Initially a 360 bp fragment is amplified using using PolF and PolR primers [34] followed by nesting with PolFI and AQER-GC30 primers [33]. To amplify the total bacterial community, the V3 variable region of 16S rRNA was amplified using V3FC and V3R primers [35]. Full PCR conditions and primer sequences are described in Orr et al, [23].

### DGGE

DGGE was carried out using the D-Code system (Bio-Rad Laboratories) as described by Baxter & Cummings [35]. PCR products were electrophoresed through gels containing 35–55% denaturing gradient at 200 V for either 6 (*nifH*) or 4.5 (16S rRNA) hours. Bands were identified and relative intensities were calculated with Quantity One software (Bio-Rad). Shannon’s diversity index (*H*′) was calculated by the formula *H*′* = -Σpi*ln(*pi*), where *pi* is the ratio of intensity of band *i* compared with the total intensity of the lane.

### qPCR

Reactions were set up using SYBR green (Thermo Fisher Scientific) according to Orr et al, [23] with the Rotor-Gene RG 3000 (Corbett Research). PolF and PolR primers were used for *nifH* qPCR, and Eub338 and V3R were used for total bacteria qPCR. A standard curve was set up using 10-fold dilutions of pGEM-T Easy vector plasmid DNA containing either the *nifH* gene of *Rhizobium* sp. strain IRBG74 bacterium [36] or the 16S rRNA gene of *Pseudomonas aeruginosa* NCTC10662. Each soil extraction, no-template control, and standard curve dilution was replicated three times. Average copy number was converted into copies of the gene per g of oven dry soil.

Standard deviation was determined (by the Rotor-Gene 6 software [Corbett Research]) on the replicate threshold cycle (*CT*) scores. qPCR was repeated if the deviation was above 0.4. Samples were considered to be below reasonable limits of detection if the *CT* score was above 30 [37]. In the system used in this study, this would equate to results below 1.0×10^4^ copies per g of soil being rejected. All no-template control results fell below this threshold (35.4±2.8). The standard curve produced was linear (*r* ^2^ = 0.98), and the PCR efficiency was 0.9.

### Sequencing

All sequencing was carried out on a 3130 genetic analyzer (Applied Biosystems). DGGE bands of interest were excised from the gel. DNA was eluted into 10 µl of sterile water and used as the template in the *nifH* PCR. The process was repeated until the band of interest was isolated. The PCR product was then cleaned up using ExoSAP-IT. PCR products were then purified using ethanol precipitation. Sequence data was analyzed using the NCBI BLAST tool.

### Statistical Analysis

In all tests, significant effects/interactions were those with a *P* value of 0.05. Treatment effects were analyzed by analysis of variance of a linear mixed effects model, using the lme function in R version 2.6.1 [38] with the maximum likelihood method and the random error term (block/year/date/crop protection) specified to reflect the nested structure of the design [39]. The combined data for all years were analyzed first, and where interaction terms were significant, further analyses were conducted at each level of the interacting factor. Where analysis at a given level of a factor was carried out, that factor was removed from the random error term. The normality of the residuals of all models was tested with QQ plots, and data were log-transformed when necessary to meet the criteria of normal data distribution. Differences between main effects were tested by analysis of variance (ANOVA). Differences between years or sample dates (both across years or within a year) were tested with Tukey contrasts in the general linear hypothesis testing (glht) function of the multcomp package in R. A linear mixed effects model was used for the Tukey contrasts, containing a year or sample date main effect with the random error term specified as described above.

Step-wise regression was carried out in Minitab [40] using the results over the three years and three sample dates for *q*PCR and Shannon’s diversity index data as response variables and the measured soil parameters listed above (pH, NO_3_^−^, NH_4_^+^, soil basal respiration, total N and organic C) as well as environmental variables (average soil temperature and total rainfall in the 14 days prior to sampling) as explanatory variables.

DGGE data were analyzed by detrended correspondence analysis (DCA) on relative intensities followed by direct ordination with Monte Carlo permutation testing. Direct ordination was either by canonical correspondence analysis (CCA) or redundancy discriminate analysis (RDA), depending on the length of the DCA axis (where an axis of >3.5 =  CCA and an axis of <3.5 =  RDA) using CANOCO 4.5 and CANODRAW for Windows [41].

## Results

### Diversity and Expression of *nifH* and 16S mRNA Transcripts and Genes

A single band of 360 bp, corresponding to the expected *nifH* mRNA transcript, was successfully amplified from RNA extracted from all 2007 and 2009 plots. However, the *nifH* mRNA transcript could not be detected in certain plots in September 2008. When using the qPCR approach the CT score for the *nifH* mRNA transcript was below the reasonable limits of detection for all plots at all sample dates in 2008. In contrast, acceptable copy numbers of the 16S mRNA transcript were successfully amplified from all 2008 samples suggesting that the *nifH* gene was not being expressed at certain dates in 2008 rather than a problem with the extraction and amplification protocol.

**Figure 1:**
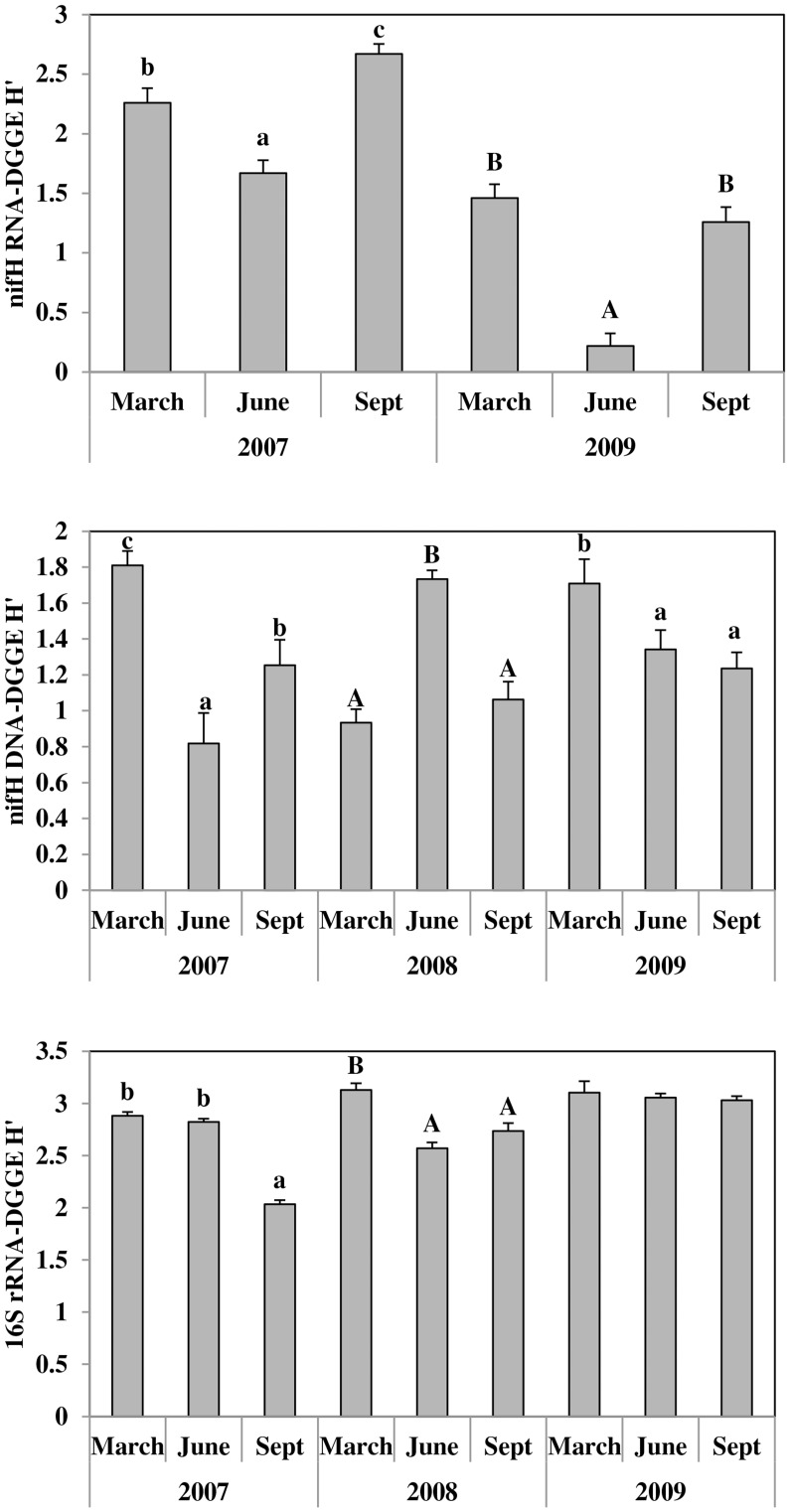
Showing Shannon’s diversity indices of the metabolically active and total diazotrophic bacteria and the total bacterial communities derived from the DGGE analyses. The *nifH* mRNA transcripts are represented by the top, the *nifH* gene by the middle and the 16S mRNA transcript by the bottom panels respectively. Bars labelled with the same letter in the same year are not different (*P* = 0.05). Bars are standard errors (n = 16).

When *nifH* RNA diversity indices (H′) from 2007 and 2009 were analyzed (Table 2), the year, sample date and sample date × year interaction terms were all significant, while crop protection and fertility management factors did not contribute to a significant proportion of the variation in results. H′ was significantly higher overall in 2007 and generally, the June sample date had the lowest diversity across the three years. However, when separate analyses were conducted for each year, sample date was highly significant for both 2007 and 2009 (P = 0.002 in both years). In both years June samples had the lowest *nifH* mRNA transcript diversity, although H′ values for June 2009 were extremely low compared with June 2007 (Fig. 1). In addition, September 2007 *nifH* mRNA transcript diversity was significantly higher than the other two sample dates in that year, whereas in 2009, there was no difference in *nifH* mRNA transcript diversity between March and September sample dates.

**Table 2:** Summary of Shannon diversity analysis of all DGGE results from all sample years and nucleic acid types.

	H′ for *nifH* DGGE (RNA) band data (mean+SE)	H′ for nifH DGGE band data (DNA) (mean+SE)	H′ for 16S rRNA DGGE band data (mean+SE)
**Year (Y)**			
** 2007**	2.20±0.08 a	1.29±0.10 a	2.58±0.06 a
** 2008**		1.24±0.07 a	2.81±0.05 a
** 2009**	0.98±0.10 b	1.43±0.07 a	3.06±0.04 b
**Sample Date (SD)**			
** March**	1.86±0.11 b	1.48±0.08^1^a	3.04±0.05 b
** June**	0.95±0.15 a	1.30±0.09 a	2.82±0.04 a
** September**	1.97±0.15 b	1.18±0.07 a	2.60±0.07 a
**Crop protection (CP)**			
** ORG**	1.62±0.13 a	1.37±0.06 a	2.82±0.05 a
** CON**	1.56±0.13 a	1.27±0.07 a	2.82±0.05 a
**Fertility management (FM)**			
** ORG**	1.57±0.13 a	1.28±0.06 a	2.79±0.05 a
** CON**	1.61±0.13 a	1.37±0.07 a	2.85±0.05 a
**ANOVA ** ***P*** **-values**			
** Y**	**0.001**		**<0.001**
** SD**	**<0.001**	**0.012**	**<0.001**
Y*SD	**0.040**	**<0.001**	**<0.001**
CP*FM			**0.006**

1 Although date was a significant factor in the ANOVA, means comparison tests did not indicate any significant differences among dates.

*P*-values are only shown for terms with *P*<0.05. Means followed by the same letter for a given factor are not significantly different (P<0.05; Tukey’s HSD test where there are more than two treatment levels).

In contrast to the RNA results, analysis of the *nifH* DNA-DGGE diversity results showed that year was not a significant factor but there was a significant interaction between sample date and year (Table 2); again, crop protection and fertility management were not significant factors in the model. Since the year × sample date term was significant, a separate analysis was conducted for each year for both RNA and DNA extractions (Fig. 1). This showed that the ranking of dates for DNA-DGGE diversity was not the same in each year. In 2007 and 2009 H′ was highest for the March sampling date, while in 2008 it was highest in June.

The diversity of the active bacterial community was also analyzed (Table 2). ANOVA indicated that overall diversity was highest in 2009, and within a given year was greatest in March; however, there were significant year by date interactions. These are illustrated in Fig. 1 which shows that sample date had no effect on bacterial community diversity in 2009, while on the other two dates there were some differences among sample dates.

**Figure 2:**
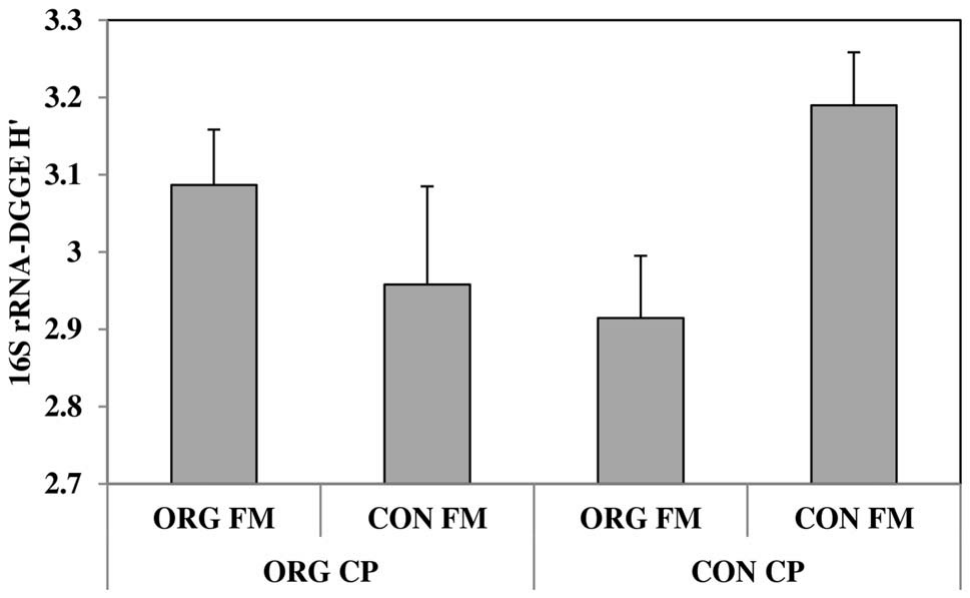
The interaction between organic and conventional crop protection (ORG CP, CON CP) and organic and conventional fertility management (ORG FM, CON FM) on March sample dates only for three years (2007, 2008 and 2009) for Shannon’s diversity index of the 16S mRNA transcript. Bars are standard errors (n = 12).

In contrast to *nifH* community diversity, the overall bacterial community diversity was affected by crop management practices. There was also a significant interaction between sample date, fertility management and crop protection. When only the March samples were analyzed across all three years, there was a significant crop protection by fertility management interaction (P = 0.007), although neither factor had a significant main effect. Fig. 2 shows that in March of every year, highest bacterial diversity was measured in the fully conventionally managed plots. However, on the other two sampling dates, crop management had no effect on bacterial community diversity and year was the only significant factor in the model. For all sample dates, highest bacterial community diversity was measured in 2009.

The diazotrophic and total bacterial community composition were further analysed using constrained ordination, for each sample date, to determine how soil biochemical properties measured on the sample dates and environmental conditions in the two weeks prior to sampling may be driving community composition. The importance of the fertility management and crop protection treatments as drivers of community composition were also investigated in the constrained ordinations. For *nifH*, although fertility management and crop protection did not represent a significant portion of the variance on any of the sample dates, factors that were significantly affected by fertility management on all sample dates did contribute significantly to the variation in *nifH* community structure. Specifically, soil basal respiration (greater under organic FM, P<0.001) and nitrate and ammonium (both greater under conventional FM, P<0.001 and  = 0.003 respectively) were correlated with changes in *nifH* diversity at certain dates. Factors associated with fertility management also contributed to a much greater proportion of the variance when analysed as separate factors rather than grouped as one management factor (Table 3). The constrained ordination did, however show that crop management significantly affected total bacterial community composition in June 2007 and June 2009 and that fertility management significantly affected total bacterial community composition in September 2008. The significant effect of fertility management in 2008 coincides with pH also significantly affecting total bacterial community composition (Table 3).

**Table 3:** Summary of CCA and RDA analysis of RNA DGGE results showing significant variables.

Gene of interest	Sample date	Variables tested	Significant variables selected by forward selection			Variance of DGGE data explained by the model (%)		
			2007	2008	2009	2007	2008	2009
*nifH*	March	FM				8.0		4.8
		CP				5.8		5.1
		Associated variables^1^				12.7		13.2
		Associated variables^1^, FM, CP				14.4		15.6
	June	FM				6.1		8.0
		CP				6.4		10.1
		Associated variables^1^			NH_4_^+^	11.1		23.6
		Associated variables^1^, FM, CP	SBR		NH_4_^+^	23.4		36
	September	FM				7.2		9.1
		CP				5.2		5.5
		Associated variables^1^			NO_3_^−^, NH_4_^+^	14.3		20.6
		Associated variables^1^, FM, CP			NO_3_^−^, NH_4_^+^	15.2		22.6
16S rRNA	March	FM				3.1	5.8	4.7
		CP				6.2	6.1	5.9
		Associated variables^1^				9.9	4.5	4.2
		Associated variables^1^, FM, CP				10	8.8	9.1
	June	FM				6.2	4.5	4.4
		CP	CP		CP	14.1	8.0	11.7
		Associated variables^1^				9.1	9.2	6.8
		Associated variables^1^, FM, CP	CP		CP	17.8	10.5	13.5
	September	FM	*FM*			11.3	9.3	5.8
		CP				4.7	3.9	5.3
		Associated variables^1^		pH	NO_3_^−^	11.2	12.9	11.2
		Associated variables^1^, FM, CP		FM, pH	NO_3_^−^	22.1	18.5	12.9

FM = fertility management, CP = crop protection.

1 Associated variables measured at the time of sampling pH, soil basal respiration (SBR), ammonium (NH_4_^+^) and nitrate (NO_3_^−^). Soil basal respiration was measured in June samples only and pH was measured in September samples only.

For the *nifH* community attempts were made to sequence all bands on the gels. This resulted in 22 bands being sequenced and identified from the DGGE gels. The sequences were around 200 bp in length and enabled gross taxonomic resolution but were too short for higher level phylogenetic affiliation to be determined. Sequence data is available at the GenBank database under accession numbers JQ618105–JQ618126. Table S2 shows the closest match from the NCBI database. Of the 22 sequences, 17 were from uncultured taxa; 10 sequences belonged to *Alpha-Proteobacteria*, 9 belonged to *Beta-Proteobacteria*, 2 belonged to *Gamma-Proteobacteria* and 1 belonged to the order Clostridia. The remaining 3 bands were identified as belonging to *Rhizobium huautlense*. By analyzing the relative intensities of the sequenced bands using ANOVA (data not shown) it was found that management type did not significantly affect the presence or intensity of any of the sequenced bands.

### Quantification of the *nifH* and 16S mRNA Transcripts and Genes with qPCR

**Figure 3:**
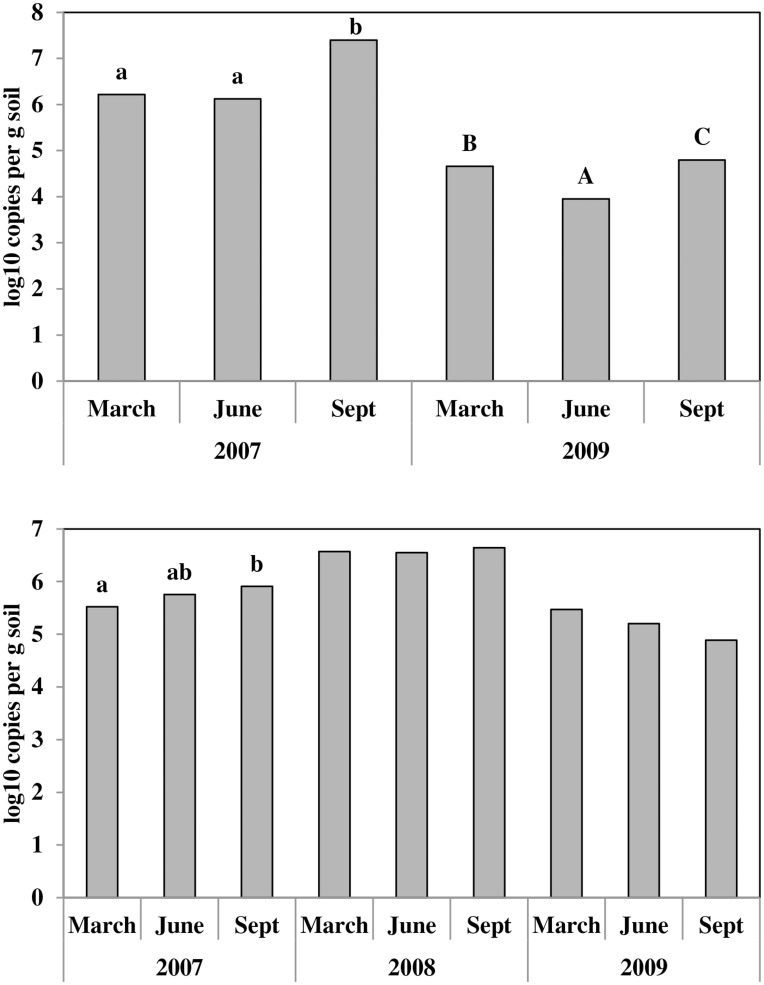
Showing copy numbers of the *nifH* mRNA transcript and the *nifH* gene. *nifH* mRNA transcripts shown in the top and *nifH* gene in the bottom panels respectively. Bars labelled with the same letter in the same year are not different (*P* = 0.05). Unlabelled bars in the same year are not significantly different.

The predominant factors affecting *nifH* mRNA transcripts and DNA copy numbers and the 16S ribosomal mRNA transcript copy numbers were Year and Sample date, although in some cases crop management practices also affected these parameters (Table 4). On average there were significantly more copies of the *nifH* mRNA transcript detected in 2007 compared with 2009. September samples also had more copies of *nifH* mRNA; however, there were strong interactions between Year and Sample date, and Year and Fertility Management. For this reason a separate year by year analysis was conducted. In both years Sample date was the predominant factor affecting *nifH* mRNA transcript copy numbers. Highest numbers were detected in September, although in 2009 significantly lower numbers were detected in June while in 2007 March and June results did not differ (Fig. 3). In both years the use of organic fertility management always resulted in higher levels of *nifH* gene expression than conventional fertilisation.

**Table 4:** Summary of qPCR analysis across all years and sample dates for all genes and nucleic acid types.

	*q*PCR average copy numbers for *nifH* RNA data set (mean ± SE)	*q*PCR average copy numbers for*nifH* DNA data set (mean ± SE)	*q*PCR average copy numbers for 16SrRNA gene data set (mean ± SE)
year (Y)			
2007	9.29×10^6^±2.85×10^6^b	5.69×10^5^±6.81×10^4^b	8.05×10^7^±1.17×10^7^a
2008		3.89×10^6^±2.62×10^5^c	5.26×10^7^±2.30×10^7^a
2009	3.92×10^4^±5.37×10^3^a	1.77×10^5^±6.34×10^4^a	2.96×10^8^±1.29×10^8^a
sample date (SD)			
March	8.46×10^5^±4.99×10^5^b	1.45×10^6^±2.65×10^5^a	2.99×10^8^±1.29×10^8^
June	6.62×10^5^±2.33×10^5^b	1.42×10^6^±2.51×10^5^a	4.31×10^7^±7.29×10^6^a
September	1.25×10^7^±4.16×10^6^a	1.76×10^6^±3.45×10^5^a	8.66×10^7^±2.41×10^7^a
Crop protection (CP)			
org	6.25×10^6^±2.72×10^5^a	1.74×10^6^±2.44×10^5^a	1.06×10^7^±5.20×10^7^a
con	3.07×10^6^±1.18×10^6^a	1.35×10^6^±2.27×10^5^b	1.81×10^8^±8.13×10^7^a
fertility management			
org	4.97×10^6^±2.59×10^6^a	1.64×10^6^±2.31×10^5^a	1.23×10^8^±3.92×10^7^a
con	4.35×10^6^±1.53×10^6^a	1.45×10^6^±2.42×10^5^	1.63×10^8^±7.99×10^7^a
ANOVA *P* values			
Y	**<0.001**	**<0.001**	
SD	**<0.001**		**0.032**
CP		**0.013**	
Y*SD	**0.005**		**<0.001**
Y*FM	**0.001**	**0.032**	
CP*FM	**0.037**		
Y*SD*FM		**0.022**	

*P*-values are only shown for terms with *P*<0.05; all data were log-transformed before analysis. Means followed by the same letter for a given factor are not significantly different (P<0.05; Tukey’s HSD test where there are more than two treatment levels).

Quantities of the *nifH* gene were also strongly affected by Year with highest copy numbers observed in 2008, but in contrast to the *nifH* mRNA transcript, crop protection practices were also significant. The use of organic crop protection practices resulted in significantly higher quantities of the *nifH* gene compared with conventional crop protection (Table 4). Year by year analysis of the *nifH* gene shows that sample date is only a significant factor in 2007 with *nifH* gene copy number increasing throughout the year (Table 5). Year by year analysis also shows that in 2007 organic fertility management results in increased *nifH* gene copy number (Table 5).

**Table 5:** Average copy numbers for *nifH* gene amplified from DNA and reverse transcribed RNA for each year of the trial.

	Copies of *nifH* RNA*/*g soil (mean±SE)		Copies of *nifH* DNA*/*g soil(mean±SE)			Copies of 16S rRNA gene/g soil(mean±SE)		
	2007	2009	2007	2008	2009	2007	2008	2009
**Sample date (SD)**								
**March**	1.65×10^6^±	4.56×10^4^±	3.31×10^5^±	3.72×10^6^±	2.94×10^5^±	6.19×10^7^±	1.66×10^7^±	8.20×10^8^±
	9.71×10^5^b	1.16×10^4^b	1.08×10^5^a	3.16×10^5^a	1.78×10^5^a	1.88×10^7^a	4.24×10^6^a	3.58×10^8^b
**June**	1.32×10^6^±	8.97×10^3^±	5.65×10^5^±	3.54×10^6^±	1.59×10^5^±	7.21×10^7^±	1.52×10^7^±	4.20×10^7^±
	4.10×10^5^b	1.13×10^2^a	1.20×10^5^ab	3.46×10^5^a	6.35×10^4^a	1.54×10^7^a	3.09×10^7^a	1.20×10^7^a
**September**	2.49×10^7^±	6.28×10^4^±	8.10×10^5^±	4.39×10^6^±	7.70×10^4^±	1.08×10^8^±	1.26×10^8^±	2.62×10^7^±
	7.15×10^6^a	5.95×10^3^c	1.00×10^5^b	6.31×10^5^a	2.47×10^4^a	2.50×10^7^a	6.65×10^7^b	4.49×10^6^a
**Crop protection (CP)**								
**ORG**	1.01×10^7^±	3.42×10^4^±	5.75×10^5^±	4.37×10^6^±	2.81×10^5^±	7.68×10^7^±	3.12×10^7^±	2.08×10^8^±
	5.00×10^6^a	6.79×10^3^a	9.46×10^4^a	2.73×10^5^a	1.23×10^5^a	1.41×10^7^a	8.73×10^6^a	1.07×10^8^a
**CON**	8.50×10^6^±	4.41×10^4^±	5.62×10^5^±	3.40×10^6^±	7.26×10^4^±	8.43×10^7^±	7.39×10^7^±	3.83×10^8^±
	2.86×10^6^a	8.33×10^3^a	1.00×10^5^a	4.30×10^5^a	1.94×10^4^a	1.90×10^7^a	4.53×10^7^a	2.36×10^8^a
**Fertility mgt (FM)**								
**ORG**	9.91×10^6^±	4.67×10^4^±	7.62×10^5^±	4.03×10^6^±	1.26×10^5^±	8.75×10^7^±	7.33×10^7^±	2.08×10^8^±
	2.76×10^6^a	8.08×10^3^a	1.07×10^5^a	3.12×10^5^a	4.32×10^4^a	1.76×10^7^a	4.53×10^7^a	1.04×10^8^a
**CON**	8.62×10^6^±	3.16×10^4^±	3.76×10^5^±	3.74×10^6^±a	2.27×10^5^±	7.36×10^7^±	3.18×10^7^±	3.83×10^8^±
	4.929×10^6^b	6.75×10^3^b	6.44×10^4^b	4.25×10^5^	1.20×10^5^a	1.57×10^7^a	8.70×10^6^a	2.37×10^8^a
**ANOVA ** ***P*** **-values**								
**SD**	**0.001**	**<0.001**	**0.050**				**0.011**	**0.009**
**FM**	**0.026**	**0.016**	**0.005**					
SD*FM					**0.036**			
SD*CP*FM							**0.048**	

*P*-values are only shown for terms with *P*<0.05; data for *nifH* RNA 2007 and 2009, *nifH* DNA 2009 only, and 16S rRNA all years, were log-transformed before analysis. Means followed by the same letter for a given factor are not significantly different (P<0.05; Tukey’s HSD test where there are more than two treatment levels).

Although the ANOVA results indicated that sample date had a significant effect on copies of the 16S mRNA transcript (Table 4) there were no significant differences among the months identified using Tukey’s HSD test. Year was not a significant factor affecting numbers of 16S mRNA transcript but a significant Year by Sample date interaction was observed. When each year was analysed individually (Table 5) it was found that Sample date was a significant factor in 2008, where highest numbers of 16S mRNA transcript were observed in September, and in 2009, where highest numbers were observed in March. Stepwise regression was used to determine how soil biochemical properties may be driving *nifH* and 16S RNA transcript and gene activity (qPCR) and diversity (DGGE H′) (Table S3). This analysis indicated that soil temperature had a slightly negative effect on *nifH* diversity (for both transcript and gene) and a positive effect on *nifH* transcript activity. Rainfall was negatively correlated with *nifH* transcript diversity (RNA) and positively related to *nifH* gene diversity. In addition, the diversity of the *nifH* mRNA transcript was negatively related to soil C and soil basal respiration. Whereas for activity of the *nifH* gene measured using DNA extracts, pH had a positive effect while soil basal respiration was negatively correlated with expression (Table S3). In general there was a positive correlation between *nifH* RNA diversity and copy number and likewise a positive correlation between 16S rRNA diversity and copy number. For the 16S rRNA gene, copy numbers were also negatively correlated with rainfall. Negative correlations were observed between the DGGE H′ data set and average soil temperature with average rainfall positively correlated with both *nifH* and 16S DNA DGGE H′. 16S rRNA DGGE H′ was also affected by available nitrate; total carbon and available ammonium. These correlations to environmental parameters are distinct from those of 16S expression, suggesting *nifH* expression did not simply mirror the response of the broader bacterial community (Table S3).

## Discussion

The NFSC trial enables studies to be conducted with spatial and temporal replication of each system of interest allowing for robust analyses of the impact of management and environmental effects on the microbial communities [42]. Previously we have shown that soils in a conventional crop rotation had a significantly greater diversity and number of free-living diazotrophic bacteria than those within an organic rotation [23]. Here we compared the effect of organic versus conventional fertility management and crop protection activities on the total and free-living N fixing bacterial communities in three different years, on three sampling dates in each year. We hypothesised that the predominant factor affecting diazotrophic and total bacterial diversity, biomass, activity and community structure would be enhanced under organic fertility management, as a result of increased levels of organic carbon, phosphorus and higher soil pH, as has been previously observed [10], [43]–[47].

However, although overall activity of soil organisms was enhanced under ORG FM (e.g. higher soil basal respiration 1.14 mg CO_2_ kg^−1^ h^−1^ for CON FM versus 1.38 mg CO_2_ kg^−1^ h^−1^ for ORG FM), and pH was significantly reduced in conventional fertility management (6.35 for CON FM versus 6.58 for ORG FM on average) while the availability of P, nitrate and ammonium was increased; (Table S4) our data demonstrated that the most significant explanatory variables for quantitative changes in both the diversity and numbers of free-living diazotrophic and total bacterial populations in agricultural soil in a multiple year study were temporal and seasonal effects. These observations contrast with previous work, where fertility source (farmyard manure versus mineral or no fertilizer) was the dominant factor driving bacterial community structure [8], [11], indicating that an increase in organic carbon, associated with organic fertility management activities, had a positive correlation with bacterial soil diversity [48], [49]. However, other studies that have more resonance with our data, indicate that changes to bacterial structure and diversity due to management practices are often subtle [50] and seasonal and plant growth effects often have a greater influence than those due to management processes [51]. One explanation for our findings may be that, although the NFSC trial has been ongoing since 2001, there were no significant differences in soil organic C or total N between the soil management treatments in any of the study years.

Although overall fertility management had no effect on the diversity of the diazotrophs, the factors soil basal respiration and available nitrate were associated with changes in *nifH* diversity and activity (Table 3). There are very few studies on the impact of organic farming on the free-living diazotrophic communities in agricultural soil. DeLuca et al, [22] compared the use of cattle manure and urea fertilizers and found that both fertilizer types inhibited nitrogen fixation (measured by acetylene reduction assay) and that pH was correlated with nitrogen fixation ability. Previous studies, looking at the effect of individual attributes of farm management on the rhizospheric nitrogen fixing community, suggested that the application of increased amounts of nitrogen fertilizer (normally associated with conventional fertility management) would result in decreased diazotrophic diversity and activity [4], [52]. Rather our data suggests that many different factors affect the nitrogen fixing community (Table 3 and S3). Although our results were not as conclusive as previous studies, organic fertility management was observed to correlate with increased *nifH* mRNA transcripts in 2007 and 2009, and increased *nifH* gene copy number in 2007 (Table 5). Soil nitrate levels were also negatively correlated with *nifH q*PCR data (Table S3), which corresponds to other studies which have reported inhibition of nitrogenase activity in free-living N_2_ fixing bacteria [53]–[56]. The interacting effects of nitrogen level, carbon availability and crop protection practices, make it difficult to recommend one suite of management practices that can be expected to enhance N fixation by diazotrophs in agricultural soils.

It was hypothesised that conventional crop protection would have a negative effect on *nifH* diversity, and expression, as studies into the environmental impacts of pesticides have shown that they can significantly affect the bacterial community as a whole and that diazotrophs could be particularly affected [57], [58], [51]. Our results suggest that conventional crop protection did in fact exert a negative effect on the diazotrophic activity when *nifH* copy numbers derived from the DNA data set were considered (Table 4) but appeared not to impact on the diversity or structure of the community. The DNA results suggest that the size of the *nifH* population in plots under conventional crop protection has been significantly reduced due to the long-term application of pesticides. That levels of *nifH* expression (RNA-qPCR results) did not mirror the DNA-qPCR results suggests that activity of the diazotrophic community is not limited by its size, but rather by other controlling factors. A range of pesticides are applied to the potato crops in the NFSC experiment (Table 1) some of which have been shown to have some inhibitory effect on diazotrophs at high concentrations [57], [59], [60]. Many previous studies looking at the effect of pesticides on the diazotrophic community have focussed on nitrogen fixers which are symbiotic with legumes. *Bradyrhizobium japonicum*, for example, has been found to be particularly susceptible to the effects of glyphosphate due to the sensitivity of its phosphate synthase enzyme [61], [62]. Other studies have found that herbicides will affect nitrogenase activity, nodule formation, nodule biomass and leghaemoglobin concentrations [61]–[63]. However, it is unclear whether this is due to direct changes in the rhizobia, indirect physiological changes in the plant, or both and does not explain why we see significant changes in the free-living nitrogen fixing community [64], [61].

In contrast crop protection strategy had no significant effect on the activity of the total bacterial population, although, it was a significant driver of community structure in June of both 2007 and 2009. To our knowledge this is the first study which fully investigates the effects of crop protection protocols in the field on the activity of both diazotrophic and total bacterial communities.

The temporal effects observed on both diversity and number of the diazotrophic, and total bacterial communities, were primarily affected by the recent environmental conditions. On most occasions, rainfall and soil temperature were significant factors affecting activity and diversity according to stepwise regression (Table S3), although the effects were not always positive. Diversity tended to be higher in March for both *nifH* (mRNA transcripts and genes) and 16S mRNA transcripts. Optimum temperature for growth and activity of diazotrophs is between 10 and 25°C (similar to the temperature range in the field between June and September) [65], [66]. Activities of the general bacterial community were largely unaffected by sample date, suggesting that this community included species with a wide range of optimal temperatures that were able to adapt to the environmental conditions throughout the growing season. As expected, diversity and copy number of the 16S rRNA gene were always higher than diversity and copy number of the *nifH* gene. Ratios of the *nifH* gene to the 16S rRNA gene (∼1 copies of *nifH* gene: 50 copies of 16S rRNA gene) were similar to ratios seen between the 16S rRNA gene and genes used in nitrogen cycling found in other studies [67], [68].

Seasonal effects observed in this study may also be related to the crop management practices that occur throughout the year. Samples taken in March are from a relatively undisturbed soil with no plant cover. June samples may be affected by frequent cultivations for weed control, especially in the organic crop protection plots. This makes it difficult to separate soil temperature and moisture effects in this study from the effects of seasonal management practices. Stage of crop growth can also influence microbial community composition. Certain members of the soil bacterial community, particularly *Acidobacteria*, *Bacteroidetes* and *Alpha*-, *Beta*-, and *Gammaproteobacteria*, have previously been observed to be diminished in summer in crop land [49]. It has been demonstrated that growth stage and seasonal effects significantly affect diversity in soil under potato and maize [69]. For example, when culture dependent and independent (cloning and DGGE) methods were used to assess bacterial diversity in bulk and rhizosphere soil in 3 species of potato, bacterial communities were observed to change as the plant developed. Higher diversity was observed around 25 days after planting, compared to growth 65 and 140 days after planting [70]. Similarly in maize, bacterial activity, as measured by PLFA and BIOLOG, changed as maize went through five leaf stage, flowering and maturity [71]. It is assumed that these observations reflect changes in the amount and quality of root exudates as the plant reaches maturity [47].

We found that management activity, temporal and seasonal factors appeared to exert no significant effect on the most abundant diazotrophs identified by sequencing the DGGE bands (Table S2). A follow up study is currently underway using pyrosequencing to more thoroughly resolve the taxonomic structure of the diazotrophic communities in these soils. Previous work looking at the impact of differing levels of nitrogen fertilization on the diazotrophic communities of soil showed that the predominant taxa were present in all soils regardless of the amounts of nitrogen fertilizer used [52], [72]. It has been suggested that the predominant taxa remain unaffected by the level of N fertilization, whereas the minor members of this community are more sensitive to such changes [73]. In conclusion we found the dominant factors affecting the diversity and numbers of both the nitrogen fixing and the total bacterial community are temporal. The only exception was the impact of conventional crop protection protocols that seemed to reduce the number of diazotrophs within the soils but not their activity. Fertility management appeared to have little effect on the diversity of both the nitrogen fixing and the total bacterial community, although soil parameters, particularly pH and the concentrations of nitrate and ammonium, were significant factors in determining community structures. The combination of our study and the work of others suggests that rather than the bacterial communities being affected directly by the nature of the fertilizers applied they are more likely to respond to changes in carbon and nitrogen levels in the soil [10], [43], [44], [74]. Although crop management practices were found to impact on the activity and function of soil bacteria, the overriding factor was consistently the year and date of sampling.

## Supporting Information

Table S1 Summary of environmental conditions measured in the experimental field during the 14 days prior to each sample date.
(DOCX)

Table S2 The closest matches for the 22 sequenced bands derived from the NCBI database.
(DOC)

Table S3 Significant explanatory variables for *nifH* and 16S rRNA gene activity (*q*PCR) and diversity (DGGE H’) determined by stepwise regression.
(DOC)

Table S4 The impact of farm management and year of sampling on environmental variables measured in each soil
(DOC)
