# Supplementary material for: Impacts of Organic and Conventional Crop Management on Diversity and Activity of Free-Living Nitrogen Fixing Bacteria and Total Bacteria Are Subsidiary to Temporal Effects
Source: PLoS One. 2012 Dec 28;7(12):e52891. doi: 10.1371/journal.pone.0052891 (PMC3532110; doi:10.1371/journal.pone.0052891)
Supplement: Table S1 — Summary of environmental conditions measured in the experimental field during the 14 days prior to each sample date. (DOCX) [file pone.0052891.s001.docx]

|  | 2007 | | | 2008 | | | 2009 | | |
| --- | --- | --- | --- | --- | --- | --- | --- | --- | --- |
|  | SD1 | SD2 | SD3 | SD1 | SD2 | SD3 | SD1 | SD2 | SD3 |
| Average Soil Temperature (°C) | 5.44 | 15.36 | 13.31 | 4.90 | 13.98 | 12.24 | 5.00 | 13.91 | 14.19 |
| Total Rainfall (mm) | 23.4 | 20.2 | 15.8 | 4.8 | 36.4 | 18.6 | 11.0 | 47.4 | 2.0 |
