# Supplementary material for: Impacts of Organic and Conventional Crop Management on Diversity and Activity of Free-Living Nitrogen Fixing Bacteria and Total Bacteria Are Subsidiary to Temporal Effects
Source: PLoS One. 2012 Dec 28;7(12):e52891. doi: 10.1371/journal.pone.0052891 (PMC3532110; doi:10.1371/journal.pone.0052891)
Supplement: Table S2 — The closest matches for the 22 sequenced bands derived from the NCBI database. (DOC) [file pone.0052891.s002.doc]

|  | | | | | | | | |
| --- | --- | --- | --- | --- | --- | --- | --- | --- |
|  | Closest match in BLAST as of 18/11/10 | E value | % max. id | Closest identified match | E value | % max. id | Closest match class | closest match order |
| *B1* | Uncultured soil bacterium clone (AY796013) | 3.00E-43 | 81% | *Bradyrhizobium japonicum* (GQ289567.1) | 9.00E-45 | 78% | *Alphaproteobacteria* | *Rhizobiales* |
| *B2* | Uncultured soil bacterium clone (EU331531) | 3.00E-22 | 81% | *Aeromonas* sp. IPPW-29 (FJ687521) | 3.00E-19 | 74% | *Gammaproteobacteria* | *Aeromonadales* |
| *B3* | Uncultured bacterium clone (HM063717) | 2.00E-33 | 84% | *Mesorhizobium alhagi* strain DB7 (GU083829.1) | 9.00E-32 | 79% | *Alphaproteobacteria* | *Rhizobiales* |
| *B4* | Uncultured bacterium clone (GQ464112.1) | 4.00E-30 | 84% | *Azospira oryzae strain* 6a3 (U97115.2) | 8.00E-26 | 81% | *Betaproteobacteria* | *Rhodocyclales* |
| *B5* | Uncultured bacterium clone (HM063793.1) | 4.80E-01 | 100% | *Desulfitobacterium hafniense* DCB-2 | 4.80E-01 | 92% | *Clostridia* | *Clostridiales* |
| *B6* | *Rhizobium huautlense* strain CCBAU 65679 (EU622086.1) | 6.00E-141 | 99% |  |  |  | *Alphaproteobacteria* | *Rhizobiales* |
| *B7* | Uncultured bacterium clone (EU241558.1) | 9.00E-44 | 79% | *Azonexus fungiphilus* strain LMG 19178 (DQ029204) | 3.00E-33 | 77% | *Betaproteobacteria* | *Rhodocyclales* |
| *B8* | Uncultured bacterium clone (HM063717.1) | 2.00E-73 | 96% | *Rhizobium etli* strain CCBAU 65830 (EU622089.1) | 1.00E-71 | 94% | *Alphaproteobacteria* | *Rhizobiales* |
| *B9* | Uncultured bacterium isolate (GU097353.1) | 3.00E-104 | 93% | *Sideroxydans lithotrophicus* ES-1 (CP001965) | 2.00E-77 | 87% | *Betaproteobacteria* | *Gallionellales* |
| *B10* | *Rhizobium huautlense* strain CCBAU 65679 (EU622086.1) | 6.00E-116 | 93% |  |  |  | *Alphaproteobacteria* | *Rhizobiales* |
| *B11* | Uncultured bacterium clone (FJ008540) | 3.00E-52 | 92% | *Azoarcus communi* strain Swub3 (U97116) | 4.00E-40 | 85% | *Betaproteobacteria* | *Rhodocyclales* |
| *B12* | *Rhizobium huautlense* strain CCBAU 65679 (EU622086.1) | 4.00E-113 | 94% |  |  |  | *Alphaproteobacteria* | *Rhizobiales* |
| *B13* | Uncultured bacterium clone (HM063739.1) | 7.00E-110 | 94% | *Rhizobium etli* strain CCBAU 65830 (EU622089.1) | 1.00E-71 | 94% | *Alphaproteobacteria* | *Rhizobiales* |
| *B14* | Uncultured bacterium clone (EU331528) | 1.00E-26 | 81% | *Azovibrio restrictus* (U97119.1) | 4.00E-11 | 74% | *Betaproteobacteria* | *Rhodocyclales* |
| *B15* | Uncultured bacterium clone (FJ263748) | 4.00E-62 | 94% | *Mesorhizobium loti* MAFF303099 (BA000012.4) | 4.00E-62 | 94% | *Alphaproteobacteria* | *Rhizobiales* |
| *B16* | Uncultured bacterium clone (HM063828.1) | 2.00E-54 | 90% | *Azoarcus communi* strain Swub3 (U97116) | 1.00E-37 | 83% | *Betaproteobacteria* | *Rhodocyclales* |
| *B17* | Uncultured bacterium clone (DQ776446.1) | 4.00E-78 | 92% | *Ideonella* Sp. Long 7 (AY231580.1) | 3.00E-44 | 91% | *Betaproteobacteria* | *Burkholderiales* |
| *B18* | Uncultured bacterium clone (HM063739.1) | 2.00E-105 | 92% | *Rhizobium etli* strain CCBAU 65830 (EU622089.1) | 2.00E-105 | 92% | *Alphaproteobacteria* | *Rhizobiales* |
| *B19* | Uncultured bacterium clone (EU331531.1) | 2.00E-105 | 82% | *Sideroxydans lithotrophicus* ES-1 (CP001965) | 2.00E-17 | 78% | *Betaproteobacteria* | *Rhodocyclales* |
| *B20* | Uncultured bacterium clone (FJ008540) | 4.00E-79 | 89% | *Pseudomonas* sp. IPPW-3 (FJ687518.1) | 5.00E-53 | 81% | *Gammaproteobacteria* | *Pseudomonadales* |
| *B21* | Uncultured bacterium clone (GU727691) | 1.00E-62 | 90% | *Bradyrhizobium* sp. MAFF 210318 (AB079620) | 3.00E-48 | 86% | *Alphaproteobacteria* | *Rhizobiales* |
| *B22* | Uncultured bacterium clone (HM063828.1) | 2.00E-54 | 90% | *Azoarcus communi* strain Swub3 (U97116) | 9.00E-38 | 83% | *Betaproteobacteria* | *Rhodocyclales* |
