# Supplementary material for: Impacts of Organic and Conventional Crop Management on Diversity and Activity of Free-Living Nitrogen Fixing Bacteria and Total Bacteria Are Subsidiary to Temporal Effects
Source: PLoS One. 2012 Dec 28;7(12):e52891. doi: 10.1371/journal.pone.0052891 (PMC3532110; doi:10.1371/journal.pone.0052891)
Supplement: Table S3 — Significant explanatory variables for nifH and 16S rRNA gene activity (qPCR) and diversity (DGGE H’) determined by stepwise regression. (DOC) [file pone.0052891.s003.doc]

| Response variable | Regression equation | R-Sq. | P value |
| --- | --- | --- | --- |
| *nifH* RNA-qPCR | - 1.46x10^7^ + 6.18x10^6^ H’ (*nifH* RNA) + 9.47x10^5^ soil temp – 1.45x10^4^ NO_3_^-^ | 27.11 | **<0.001** |
| *nifH* DNA-qPCR | - 1.11x10^7^ + 2.10 x10^6^ pH – 9.41x10^5^ SBR | 56.6 | **<0.001** |
| 16S rRNA-qPCR | 1.10x10^7^ – 1.20x10^7^ rain fall + 4.17 x10^8^ H’ (16S rRNA) | 8.72 | 0.117 |
| *nifH* RNA DGGE H’ | 5.62 + 1.00x10^-6^ qPCR (*nifH* RNA) - 1.37 %C - 0.395 SBR - 0.0569 soil temp - 0.0164 rain fall | 60.33 | **<0.001** |
| *nifH* DNA DGGE H’ | 2.30 - 0.0680 soil temp + 0.0256 rain fall | 19.21 | **0.001** |
| 16S rRNA DGGE H’ | 1.90 + 0.378 %C - 0.0110 NH_4_^+^ + 0.00181 NO_3_^-^ - 0.0709 soil temp + 0.0107 rain fall + 1.00x10^-6^ qPCR (16 S rRNA) | 51.05 | **<0.001** |
