# Supplementary material for: Impacts of Organic and Conventional Crop Management on Diversity and Activity of Free-Living Nitrogen Fixing Bacteria and Total Bacteria Are Subsidiary to Temporal Effects
Source: PLoS One. 2012 Dec 28;7(12):e52891. doi: 10.1371/journal.pone.0052891 (PMC3532110; doi:10.1371/journal.pone.0052891)
Supplement: Table S4 — The impact of farm management and year of sampling on environmental variables measured in each soil (DOC) [file pone.0052891.s004.doc]

|  | | **Total C** | **Organic N** | **pH** | **SBR (mg CO_2_ kg^-1^ h^-1^)^z^** | **P** | **Fe** | **Available NO_3_^-^** | **Available NH_4_^+^** | **Average rainfall 2 weeks prior to sampling** | **Average soil temp 2 weeks prior to sampling** |
| --- | --- | --- | --- | --- | --- | --- | --- | --- | --- | --- | --- |
|  | | **(%)** | **(%)** |  |  | **(mg kg^-1^)** | **(mg kg^-1^)** | **(kg ha^-1^)** | **(kg ha^-1^)** | **mm** | **°C** |
| **Year** | |  |  |  |  |  |  |  |  |  |  |
| **2007** | | 1.8 ± 0.14 c | 0.3 ± 0.04 **a** | 6.27 ± 0.2 *b* | 1.24 ± 0.4 b | 51.3 ± 3.7 y | 330 ±11 **y** | 104.07 ± 22.9 *x* | 4.97 ± 0.5 x | 1.41 ± 0.0 d | 11.37 ± 0.0 **d** |
| **2008** | | 2.7 ± 0.15 a | 0.2 ± 0.02 **b** | 6.80 ± 0.3 *a* | 0.86 ± 0.1 c | 48 ± 4.2 y | 373 ± 17 **y** | 29.24 ± 6.4 *y* | 2.05 ± 0.5 x | 1.42 ± 0.0 d | 10.37 ± 0.0 **d** |
| **2009** | | 2.2 ± 0.15 b | 0.2 ± 0.02 **c** | 6.31 ± 0.3 *b* | 1.89 ± 0.5 a | 63.2 ± 2.5 x | 412 ± 13 **x** | 85.22 ± 19.8 *xy* | 6.20 ± 1.8 x | 2.09 ± 0.6 d | 11.03 ± 0.7 **d** |
| **sample date** | |  |  |  |  |  |  |  |  |  |  |
| **March** | |  |  |  |  |  |  | 3.71 ± 0.5 h | 0.84 ± 0.2 **h** | 0.93 ± 0.4 *h* | 5.11 ± 0.2 i |
| **June** | |  |  |  |  |  |  | 195.53 ± 23.2 g | 9.37 ± 1.7 **g** | 2.48 ± 1.2 *g* | 14.42 ± 0.5 g |
| **September** | |  |  |  |  |  |  | 19.29 ± 2.0 h | 3.02 ± 0.5 **h** | 1.23 ± 0.8 *gh* | 13.25 ± 0.3 h |
| **Crop prot** | |  |  |  |  |  |  |  |  |  |  |
| **ORG** | | 2.3 ± 0.05 | 0.2 ± 0.00 | 6.51 ± 0.0 | 1.27 ± 0.1 | 53.4 ± 1.7 | 378 ± 8 | 75.91 ± 15.7 | 4.46 ± 0.9 |  |  |
| **CON** | | 2.3 ± 0.05 | 0.2 ± 0.00 | 6.41 ± 0.0 | 1.25 ± 0.1 | 54.9 ± 1.9 | 364 ± 7 | 69.78 ± 14.3 | 4.36 ± 0.9 |  |  |
| **Fert. Manag.** | |  |  |  |  |  |  |  |  |  |  |
| **ORG** | | 2.5 ± 0.05 | 0.2 ± 0.00 | 6.58 ± 0.0 | 1.38 ± 0.7 | 50.6 ± 1.6 | 368 ± 8 | 32.88 ± 5.1 | 2.76 ± 0.3 |  |  |
| **CON** | | 2.3 ± 0.05 | 0.2 ± 0.00 | 6.35 ± 0.0 | 1.14 ± 0.1 | 57.7 ± 1.9 | 376 ± 8 | 112.81 ± 19.5 | 6.06 ± 1.3 |  |  |
| **ANOVA** *P*-values | |  |  |  |  |  |  |  |  |  |  |
| **Year** | | **<0.001** | **<0.001** | **<0.001** | **<0.001** | **<0.001** | **<0.001** | **<0.001** | **0.034** | 0.684 | **<0.001** |
| **sample date** | |  |  |  |  |  |  | **<0.001** | **<0.001** | **0.044** | **<0.001** |
| **CP** | | 0.202 | 0.617 | **0.038** | 0.825 | 0.221 | 0.459 | 0.580 | 0.965 |  |  |
| **FM** | | 0.419 | 0.450 | **<0.001** | **<0.001** | **0.004** | 0.528 | **<0.001** | **0.003** |  |  |
|  |  | |  |  |  |  |  |  |  |  |  |
